# Bone Marrow Mesenchymal Stromal Cells on Silk Fibroin Scaffolds to Attenuate Polymicrobial Sepsis Induced by Cecal Ligation and Puncture

**DOI:** 10.3390/polym13091433

**Published:** 2021-04-29

**Authors:** Ok-Hyeon Kim, Jun-Hyung Park, Jong-In Son, Ok-Ja Yoon, Hyun-Jung Lee

**Affiliations:** 1 Department of Anatomy and Cell Biology, College of Medicine, Chung-Ang University, Seoul 06974, Korea; ssimba315@cau.ac.kr (O.-H.K.); june8902@cau.ac.kr (J.-H.P.); jison@cau.ac.kr (J.-I.S.); 2 Department of Global Innovative Drugs, Graduate School of Chung-Ang University, Seoul 06974, Korea; 3 Da Vinci College of General Education, Chung-Ang University, Seoul 06974, Korea

**Keywords:** mesenchymal stem cells, silk fibroin nanofiber, sepsis, inflammation, scaffold

## Abstract

Suitable scaffolds with appropriate mechanical and biological properties can improve mesenchymal stromal cell (MSC) therapy. Because silk fibroins (SFs) are biocompatible materials, they were electrospun and applied as scaffolds for MSC therapy. Consequently, interferon (IFN)-primed human bone marrow MSCs on SF nanofibers were administered into a polymicrobial sepsis murine model. The IL-6 level gradually decreased from 40 ng/mL at 6 h after sepsis to 35 ng/mL at 24 h after sepsis. The IL-6 level was significantly low as 5 ng/mL in primed MSCs on SF nanofibers, and 15 ng/mL in primed MSCs on the control surface. In contrast to the acute response, inflammation-related factors, including HO-1 and COX-2 in chronic liver tissue, were effectively inhibited by MSCs on both SF nanofibers and the control surface at the 5-day mark after sepsis. An in vitro study indicated that the anti-inflammatory function of MSCs on SF nanofibers was mediated through enhanced COX-2-PGE_2_ production, as indomethacin completely abrogated PGE_2_ production and decreased the survival rate of septic mice. Thus, SF nanofiber scaffolds potentiated the anti-inflammatory and immunomodulatory functions of MSCs, and were beneficial as a culture platform for the cell therapy of inflammatory disorders.

## 1. Introduction

Sepsis is a life-threatening, multi-organ dysfunction caused by a dysregulated host response to microbial infections [1]. Owing to an increase in multidrug-resistant bacterial strains, most antimicrobials are inadequate at reducing inflammatory responses. While organ support systems, such as ventilators and dialysis, are available extensively in intensive care units, the morbidity and mortality related to severe inflammatory disease remain significant [2]. Despite the continuous development of therapeutic strategies, sepsis remains a significant clinical problem and the leading cause of death in critically ill patients [3,4]. Thus, the use of mesenchymal stromal cells (MSCs) has been recognized as a promising therapeutic strategy for the treatment of sepsis, owing to its immunomodulatory and anti-inflammatory properties [5]. Recent studies have shown that MSCs have a multimodal mechanism of action, involving cytokines and other factors, the secretion of extracellular vesicles, and even cell–cell contact-dependent processes [6,7]. Additionally, the application of MSCs to sepsis has been shown to improve the outcome of sepsis in different pathologies [8,9].

Despite the effectiveness of MSCs in treating sepsis, challenges remain in creating MSC doses at the enough scale to facilitate extensive clinical studies. Currently, the isolation of MSCs from various tissue sources is based on plastic adherence, and is characterized by standard surface markers using flow cytometry and multilineage differentiation assays [10]. This process results in populations of cells with different differentiation capacities, and requires significant research time and resources in order to generate a sufficient dose for actual application.

The preconditioning of cells ex vivo, in a specifically engineered environment with different physical or chemical parameters, is one approach to improve the ability of MSCs to enhance immune response regulation. Studies have shown that MSCs can differentiate into several specialized cell types, such as adipocytes, osteoblasts, and chondrocytes [11], and enhance their immunomodulatory functions [12] when stimulated with the appropriate signals. Additionally, biomimetic mechanical stimuli or topographical cues can also improve the immunomodulatory and anti-inflammatory properties of MSCs [12,13].

Silk proteins are potential biomaterials for tissue engineering applications because of their similarity to the native extracellular matrix (ECM), their availability and the ability to process them into various material forms easily, and their ability to support the attachment of different cell types [14,15]. Our previous study showed that MSCs preconditioned with interferon gamma (IFN-γ) showed higher anti-inflammatory properties on the silk fibroin (SF) nanofiber scaffolds than they did on the poly(lactic-co-glycolic acid) (PLGA) nanofiber scaffolds [13], suggesting that in addition to biomimetic structures, material properties are key factors that increase the immunomodulatory function of MSCs. In this study, we investigated whether primed MSCs on SF nanofibers show enough potential to protect mice from polymicrobial sepsis, which is a promising culture platform to enhance the immunomodulatory properties of MSCs. In this study, electrospun SF nanofibers were used as scaffolds for MSC therapy. MSCs were cultivated on the SF nanofiber scaffolds or on glass surfaces, before being administered to the polymicrobial sepsis animal model in order to investigate whether SF nanofiber scaffolds serve as a promising culture platform for MSC therapy to treat inflammatory disorders. While the SF nanofiber scaffolds provide a beneficial microenvironment for MSCs to enhance prostaglandin E_2_ (PGE_2_) secretion and reduce the increase in plasma IL-6 levels, chronic conditions showed comparable outcomes. The expression of inflammation-related factors, such as HO-1 and COX-2, in liver tissue was effectively downregulated by both sets of MSCs, regardless of the surface.

## 2. Materials and Methods

### 2.1. Preparation of Silk Solution

The degumming method was used to extract sericin from cocoons in a silk solution by soaking in 1 g/L of 0.02 M Na_2_CO_3_ (Sigma Aldrich, St. Louis, MO, USA) at 85 °C for 1 h. The cocoons were then rinsed several times with distilled water, and the silk fibers were dried for 24 h at 37 °C. The degummed silk fibers were dissolved in 9.3 M LiBr (Sigma Aldrich, St. Louis, MO, USA) at 60 °C for 6 h. This solution was dialyzed against distilled water for 2 days using a dialysis membrane (molecular weight cut-off of 12 kDa, Sigma Aldrich, St. Louis, MO, USA) for salt removal. The dialyzed solution was filtered and lyophilized to obtain the regenerated SF solids. The final concentration of the silk fibroin solution was 5 wt% [16]. An 18 wt% SF solution was dissolved with the regenerated SF solid in formic acid (98%, Sigma Aldrich, St. Louis, MO, USA), and dissolved by magnetic stirring for 3 h.

### 2.2. Fabrication and Characterization of SF Nanofibers

The prepared SF solution was electrospun by placing it in 10 mL syringes with a 21G needle. A syringe was pumped at a rate of 0.1 mL/h to supply the solution to the needle tip. An electrical potential of 23 kV was applied between the needle tip (anode) and the collection plate (cathode), placed at a distance of 17 cm. The electrospun SF nanofiber scaffolds were formed on the surface of a glass coverslip (with a diameter of 25 mm) on the collection plate, by evaporating the solution, and because of the effect of the electric field.

The surface chemical properties of the SF nanofibers were analyzed using X-ray photoelectron spectroscopy (XPS; PHI Quantera-II, Physical Electronics Inc., Chigasaki, Japan). XPS spectra were measured using a pass energy of 20 eV with non-monochromatic AlKα radiation (195 W) over a wide scan range (1–1000 eV). The hydrocarbon C1s peak at a binding energy of 285 eV was used as a reference for the core-level signals. The spectral analysis of peak shapes was performed using Gaussian fitting. Chemical and structural analyses of the SF nanofibers were performed using Fourier-transform infrared spectrophotometry (FTIR; Nicolet 6700, Thermo Fisher Scientific, Waltham, MA, USA). The FTIR spectra were obtained via 32 scans with a resolution of 4 cm^−1^ in the frequency range of 4500–650 cm^−1^. Owing to the difficulty in measuring the transmittance of the SF nanofibers on the coverslip, FTIR analysis was performed with the SF film having identical concentration as the SF nanofibers. The morphology of the SF nanofiber scaffold was measured using field-emission scanning electron microscopy (FE-SEM; JSM 7500F, JEOL Ltd., Akishima, Tokyo, Japan).

### 2.3. MSC Preparation

Human bone marrow MSCs were derived from the whole bone marrow of independent human donors (commercially available from AllCells, Alameda, CA, USA). The cells were cultured in modified Eagle’s medium α (Thermo Fisher Scientific, Waltham, MA, USA) supplemented with 20% fetal bovine serum (Thermo Fisher Scientific, Waltham, MA, USA) and 1% penicillin and streptomycin (Thermo Fisher Scientific, Waltham, MA, USA) in a humidified atmosphere of 5% CO_2_ at 37 °C. The MSCs were cultured on the control surface or the SF nanofibers until approximately 90% confluence, and then treated with 100 ng/mL human IFN-γ (Peprotech, Rocky Hill, NJ, USA).

### 2.4. Animal Surgery

Female C57BL/6 mice (18–20 g) were purchased from DBL (Eumseong-gun, Chungcheongbuk-do, Korea), caged at a density of 5 mice per cage, and maintained on a normal laboratory diet and tap water ad libitum in an air-conditioned room (21 ± 2 °C) with a 12 h light–dark cycle. The experimental procedures were approved by the Institutional Animal Care Use Committee (IACUC) of Chung-Ang University (Approval ID: 201900071). The mice were anesthetized briefly with 0.2 mg/kg Avertin. The abdominal zone was shaved and prepared with 70% ethanol. For cecal ligation and puncture (CLP), a ventral midline incision was made, and the cecum was ligated ~2 cm distal to the ileocecal valve using silk 2-0 sutures. The ligated cecum was punctured with a 21G needle, gently squeezed to express a small amount of feces, and then returned to the central abdominal cavity. The animals in the sham group underwent the same procedures, except that the cecum was neither ligated nor punctured. The abdominal cavity was closed with silk 4-0 sutures in two layers: the abdominal wall, and the skin. Immediately after surgery, the animals were subcutaneously injected with 1 mL saline and 25 mg/kg imipenem (Sigma Aldrich, St. Louis, MO, USA) for fluid resuscitation and infection prevention. Six hours after the CLP procedure, the MSCs were detached from the SF nanofiber or control surfaces with trypsin-EDTA (Thermo Fisher Scientific, Waltham, MA, USA), and either 10^6^ MSCs in 0.2 mL PBS, or only 0.2 mL of PBS, were slowly administered through the tail vein. The mice were divided into five groups: (1) the sham control group; (2) the vehicle group; (3) the MSC group (100 ng/mL IFNγ treated MSCs on the control surface); (4) the SF–MSC group (100 ng/mL IFNγ treated MSCs on the SF nanofibers); and (5) SF–MSC plus indomethacin group (100 ng/mL IFNγ and 100 μM indomethacin-treated MSCs on the SF nanofibers).

### 2.5. Measurement of Prostaglandin E_2_ (PGE_2_) Production

Human bone marrow-derived MSCs were seeded onto the control surface and the SF nanofiber-coated surface, attached to a 6-well plate until ~90% confluence, and treated with 100 ng/mL IFNγ or 100 ng/mL IFNγ and 100 μM indomethacin. After 18 h of incubation at 37 °C, the supernatant was collected and analyzed for PGE_2_ using a PGE_2_ ELISA kit (R&D Systems Inc., Minneapolis, MN, USA).

### 2.6. Determination of Serum Cytokine Levels

The distal tail was clipped, and blood was drawn into a pipette tip in order to collect the peripheral blood. The samples were centrifuged (3000 rpm, 15 min, 4 °C), and the serum was stored at −80 °C. The concentrations of IL-6 and tumor necrosis factor-α (TNF-α) were measured using ELISA kits (Abfrontier, Seoul, Korea) according to the instructions provided by the manufacturer.

### 2.7. Hematoxylin–Eosin (H&E) Staining Assay

Liver tissues were fixed in 10% neutral buffered formalin (Biosesang, Seongnam-si, Gyeonggi-do, Korea), dehydrated, and embedded in paraffin blocks. All tissues were sectioned into thin pieces (4 μm in thickness), fixed on a glass slide, dried, and stained. The sections were soaked in xylene, gradient concentrations of ethanol, hematoxylin, and eosin, and then mounted according to the manufacturer’s instructions. They were then dried, observed, and photographed using an inverted microscope (Leica Microsystems, Wetzlar, Hesse, Germany) equipped with a Leica camera (Leica).

### 2.8. RNA Extraction and Quantitative Real-Time PCR

Total RNA was extracted from liver tissues using TRIzol (Thermo Fisher Scientific, Waltham, MA, USA). Reverse transcription of RNA was performed using a Maxima First Strand cDNA Synthesis Kit (Thermo Fisher Scientific, Waltham, MA, USA). Quantitative real-time polymerase chain reaction (PCR) was performed using the Power SYBR Green PCR Master Mix reagent (Applied Biosystems, Foster city, CA, USA) and QuantStudio 3 Real-Time PCR (Applied Biosystems, Foster city, CA, USA). The sequences for primers of Real-Time PCR are summarized as follows: IDO-1 (forward 5′-CGG ACT GAG AGG ACA CAG GTT AC-3′ and reverse 5′-ACA CAT ACG CCA TGG TGA TGT AC-3′), COX-2 (forward 5′-GCT TCA AAC AGT TTC TCT ACA ACA A-3′ and reverse 5′-CAT TTC TTC CCC CAG CAA C-3′), HO-1 (forward 5′-TGC TAG CCT GGT GCA AGA TA-3′ and reverse 5′-GCC AAC AGG AAG CTG AGA GT-3′), GAPDH (forward 5′-TGT GTC CGT CGT GGA TCT GA-3′ and reverse 5′-CCT GCT TCA CCA CCT TCT TGA-3′). The cycle thresholds (Ct) were determined using the QuantStudio Design & Analysis software v1.4.3 (Applied Biosystems to calculate the fold change), and the mRNA expression was normalized to that of GAPDH.

### 2.9. Western Blotting

Liver tissues were lysed in RIPA buffer (Thermo Fisher Scientific, Waltham, MA, USA) containing a 1% protease inhibitor cocktail and a phosphatase inhibitor cocktail (GenDEPOT, Katy, TX, USA). Total protein concentrations were quantified using a Bradford assay kit (Bio-Rad, Hercules, CA, USA). Equal amounts of proteins were separated by SDS-PAGE and analyzed using the immunoblotting method. Western blotting was performed using standard procedures with rabbit anti-cyclooxygenase-2 (COX-2, Abcam, Cambridge, UK, ab102005), rabbit anti-heme oxygenase-1 (HO-1, Abcam, Cambridge, UK, ab13243), and GAPDH (Santa Cruz Biotechnology, Dallas, TX, USA, sc-47778). Gel images were captured using the LAS 4000 (GE Healthcare Life Sciences, Chicago, IL, USA).

### 2.10. Survival Studies

Survival was assessed every 6 h within the first 24 h after surgery, and then every 12 h for 4 days. All of the mice were euthanized at the end of the fifth day. Survival curves were analyzed using the log-rank analysis and GraphPad Prism 9 software (GraphPad Inc., San Diego, CA, USA).

### 2.11. Statistical Analyses

All data are expressed as the mean ± S.E.M. Group results were analyzed using one-way analysis of variance (ANOVA) and Tukey’s method for multiple comparisons using the GraphPad Prism 9 software (GraphPad Inc., San Diego, CA, USA). Significant differences between the groups were established at * *p* < 0.05, ** *p* < 0.01, *** *p* < 0.001, and **** *p* < 0.0001.

## 3. Results

### 3.1. Characterization of the SF Nanofiber Scaffolds

**Figure 1:**
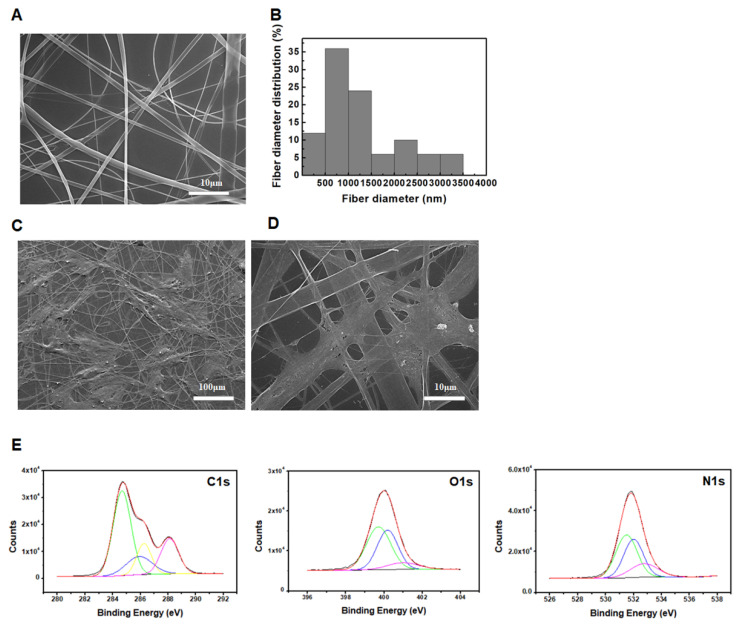
Properties and characterization of the electrospun SF nanofibers. (**A**) Scanning electron microscope (SEM) images of the SF nanofibers. Scale bar = 10 μm. (**B**) Histograms depicting the diameter distribution of the SF nanofibers. (**C**,**D**) Human bone marrow-derived MSCs on the SF nanofibers fixed in 2% glutaraldehyde, and ultrastructural images obtained by SEM. Scale bar = 100 μm, 10 μm. (**E**) X-ray photoelectron spectroscopy (XPS) analysis of the electrospun SF nanofibers.

In our previous study, the molecular weight of silk fibroin was estimated to be approximately 23 kDa. The same silk fibroin was then electrospun to generate nanofibers and analyze them, as shown in Figure 1. The average nanofiber diameter was found to be 1329.94 ± 838.31 nm (*n* = 50), and the diameter distribution of the fibers is described in the histogram in Figure 1B. After generating mesh scaffolds using the SF nanofibers, human bone marrow-derived MSCs were seeded onto the scaffold or glass surface (cell culture treated), and ultrastructural analysis was performed using scanning electron microscopy (SEM). The MSCs were attached and tightly integrated with the SF nanofibers (Figure 1C,D).

The C1s, O1s, and N1s peaks of the electrospun SF nanofibers, as identified by XPS, are shown in Figure 1C, and the atomic concentrations of C, O, and N on each sample’s surface were 22.6%, 64.66%, and 12.74%, respectively. The C1s regions of the electrospun SF nanofibers consist of four components: 284.7 eV (C–C and C=C), 285.9 eV (C–C and C–H), 286.3 eV (C–O), and 288.1 eV (C=O). The three component bands of the O1s spectrum appeared at 531.4 eV (C–O), 531.9 eV (O=CN), and 532.8 eV (C–OH/C–O–C). The three component bands of the N1s spectrum appeared at 399.7 eV (C–NH), 400.0 eV (C–N), and 401.0 eV (NH_2_) [17,18]. The above XPS data indicate the presence of functional groups, such as ether (C=O), amide (O=CN), amine (NH_2_), and hydroxy (–OH) groups on the electrospun SF nanofiber surfaces. The FTIR spectra of SF nanofibers are shown in Figure S1. The features at a wave number of 3342 cm^−1^ correspond to NH and OH stretching vibration, those at 2987 and 2913 cm^−1^ correspond to C–H stretching, and those at 1451, 1415, and 1336 cm^−1^ correspond to CH_3_ bending. The peaks at 1171 and 1073 cm^−1^ were assigned to C–O stretching. The peak at 1648 cm^−1^ (random coil) indicates amide I (C–N bonding), the band at 1543 cm^−1^ indicates amide II (secondary NH bending), and the band at 1246 cm^−1^ indicates amide III (C–C–N bending). The secondary structure of *B. mori* silk fibroin consists of the major conformations: random coils (silk I), and β-sheet (silk II). The random coil conformation of silk fibroin showed strong absorption bands at 1665 (amide I), 1540 (amide II), and 1235 cm^–1^ (amide III), while the β-sheet conformation showed absorption bands at 1628 (amide I), 1533 (amide II), and 1265 cm^–1^ (amide III) [19]. The peak at 1246 cm^−1^ indicates the β-sheet conformation, and strong bands at 1543 and 1648 cm^−1^ are attributed to the random coil conformation of SF nanofibers, as reported in [19,20].

### 3.2. MSCs on the SF Nanofibers Reduce the IL-6 Level Efficiently in Septic Mice

**Figure 2:**
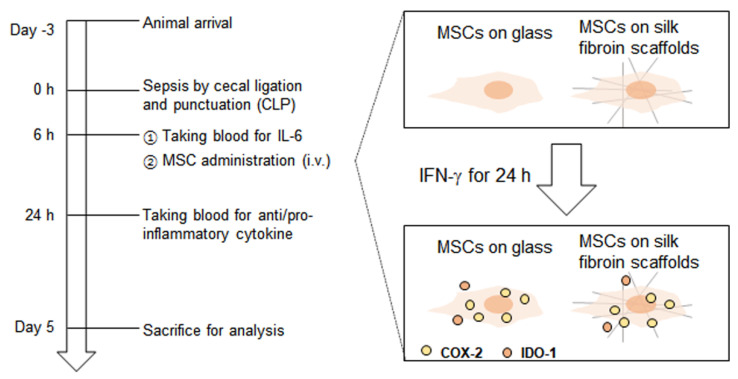
Representation of the experimental scheme.

**Figure 3:**
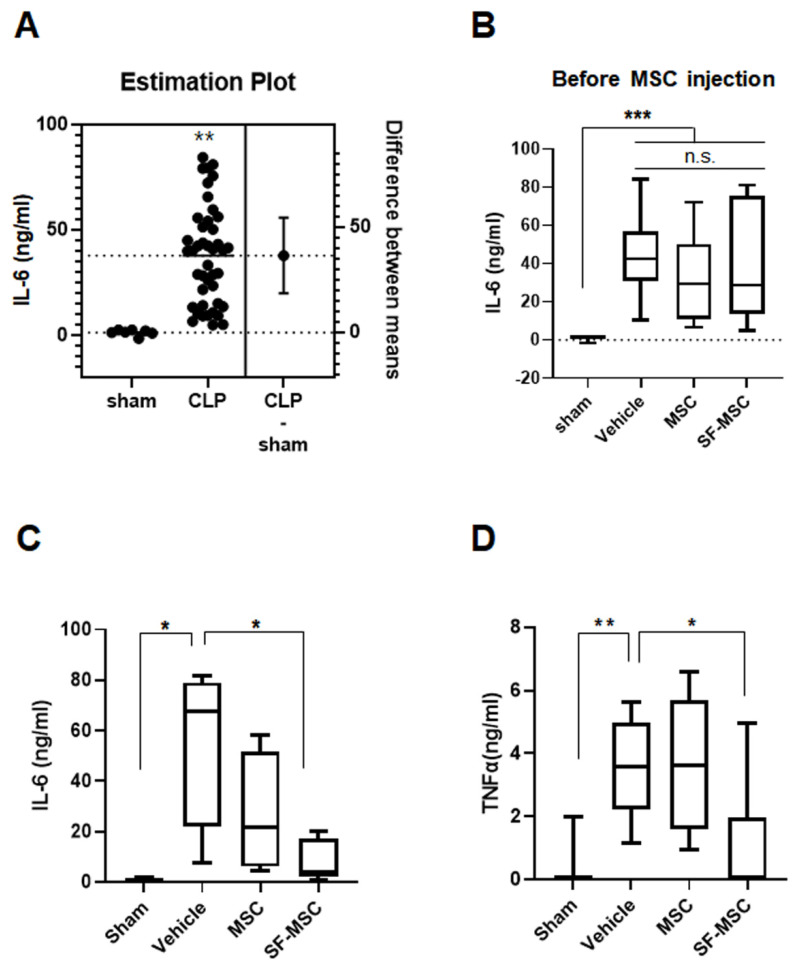
Primed MSCs on the SF nanofibers suppress the level of inflammatory cytokines in septic mice. (**A**) Plasma IL-6 levels were elevated in the CLP mice (*n* = 7 animals in the sham group vs. *n* = 43 animals in the CLP group, unpaired *t*-test, *p* = 0.0006). (**B**) IL-6 levels did not differ between the groups before the administration of MSCs (1.28 ng/mL in sham, 43.72 ng/mL in CLP group before injecting vehicle, 32.65 ng/mL in CLP group before injecting primed MSCs on the control surface, 37.77 ng/mL in CLP group before injecting primed MSCs on the SF nanofibers, one-way ANOVA, *** *p* < 0.001). (**C**) IL-6 levels were significantly reduced in the septic mouse group injected with primed MSCs on the SF nanofibers at 24 h (0.557 ng/mL in sham, 34.50 ng/mL in CLP group, 16.14 ng/mL in CLP with primed MSCs on the control surface, 6.458 ng/mL in CLP with primed MSCs on the SF nanofibers, one-way ANOVA, and * *p* < 0.05). (**D**) TNF-α levels were highly enhanced in the CLP mice, while effectively attenuated by primed MSCs on the SF nanofibers (0.2843 ng/mL in sham, 3.501 ng/mL in CLP group, 3.5412 ng/mL in the CLP with primed MSCs on the control surface, 1.006 ng/mL in the CLP with primed MSCs on the SF nanofibers, one-way ANOVA, * *p* < 0.05, and ** *p* < 0.01). Abbreviation: MSC, primed MSCs on the control surface; SF_MSC, primed MSCs on the SF nanofibers; n.s., not significant.

In our previous study, we found that IFN-γ-primed MSCs on the SF nanofibers showed higher levels of IDO-1 and COX-2 expression, and effectively suppressed T-cell activation from LPS compared to primed MSCs on the PLGA nanofibers [13]. Thus, primed MSCs on the SF nanofibers were applied to a polymicrobial sepsis murine model, in order to determine whether the SF nanofiber scaffolds have the potential to provide the required conditions for MSC therapy. As shown in Figure 2, 100 ng/mL IFNγ was applied as a priming process to MSCs on either the control surface or the SF nanofiber surface. The next day, the polymicrobial sepsis murine model was induced by the CLP, as described thoroughly in the Materials and Methods section. At 6 h after sepsis, plasma IL-6 levels were determined to be 40 ng/mL, as shown in Figure 3A. These mice were then randomly grouped as: injection of vehicle, MSCs on the SF nanofibers, or MSCs on the control surface—as shown in Figure 3B. Primed MSCs were trypsinized from either the SF nanofibers or the control surfaces, and administered through the tail veins of septic mice, with 10^6^ cells per mouse. At 18 h after MSC injection, the plasma IL-6 level was reduced by 34.5 ± 13.55 ng/mL in the vehicle group, whereas MSCs on the control surface were recorded as 16.14 ± 8.14 ng/mL IL-6, and MSCs on the SF nanofibers were determined to be 6.45 ± 1.93 ng/mL IL-6 (Figure 3B). Moreover, MSCs on the SF nanofibers significantly suppressed pro-inflammatory cytokines, and TNF-α levels were found to be 1.007 ± 0.634 ng/mL lower than those in the vehicle group (3.501 ± 0.473 ng/mL) or the MSCs on the control surface group (3.541 ± 0.574 ng/mL) (Figure 3D), suggesting that priming MSCs on the SF nanofibers effectively mitigated the inflammatory response in polymicrobial septic mice shortly after administration.

MSCs were pre-incubated with 100 ng/mL human IFN-γ for 24 h before administration. The cecum of the C57BL/6 mice was ligated and punctuated with a 21G needle for polymicrobial sepsis. Six hours after the CLP, the mouse blood was collected from their tails in order to assess IL-6 levels. Primed MSCs on either the SF nanofibers or the control surfaces were intravenously administered to the septic mice with 10^6^ cells. At 24 h after CLP, the plasma IL-6 levels were estimated. All groups were monitored for 5 days and sacrificed for biochemical analysis.

### 3.3. Abrogation of Cecal Ligation and Puncture Induced Inflammation by MSCs on SF Nanofibers

**Figure 4:**
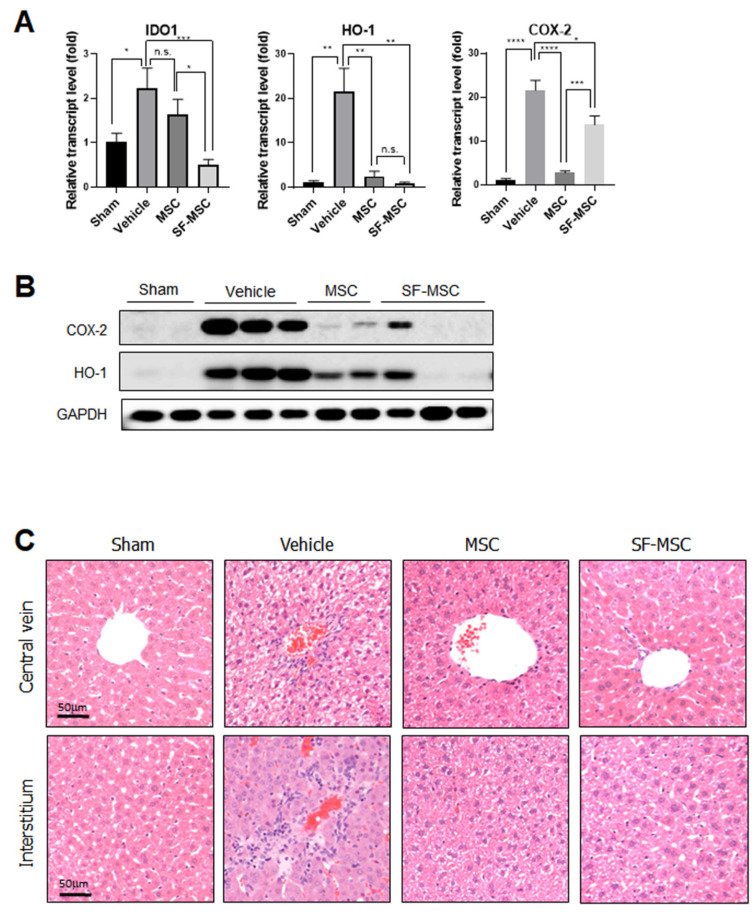
Primed MSCs on both the control surface and the SF nanofibers ameliorate inflammation-related protein expression in septic mice. (**A**) Liver tissue was isolated for the qPCR analysis of the mice’s IDO-1, HO-1 and COX-2 genes (*n* = 4 independent experiments, one-way ANOVA, * *p* < 0.05, ** *p* < 0.01, *** *p* < 0.001, and **** *p* < 0.0001). (**B**) HO-1 and COX-2 protein expression were assessed in the mice’s liver tissue 5 days after sepsis (*n* = 3 independent experiment). (**C**) The H&E staining method was applied on day 5 after sepsis. Scale bar = 50 μm.

To evaluate the protective effect of primed MSCs on the SF nanofibers under chronic conditions, liver tissue from the septic mice was isolated on day 5 in order to determine the expression levels of inflammatory response-related molecules, including IDO-1, HO-1, and COX-2. As shown in Figure 4A, the IDO-1 gene was highly enhanced in the liver on day 5 after sepsis. Although primed MSCs on the control surface could not reduce IDO-1 expression, the injection of cells from the SF nanofibers significantly blocked IDO-1 induction by sepsis. Both the gene and protein expression of HO-1 and COX-2 were dramatically attenuated by primed MSCs, regardless of the culture surface (Figure 4A,B). Moreover, primed MSCs protected against severe morphological changes and cell death in the liver tissue of polymicrobial septic animals (Figure 4C).

### 3.4. MSCs on SF Nanofibers Produce More PGE_2_

**Figure 5:**
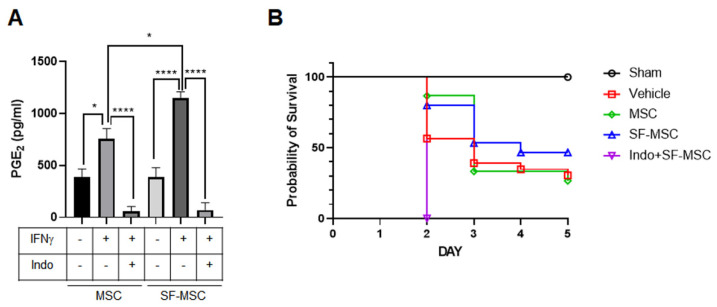
Inhibiting PGE_2_ secretion completely fails to save mice from septic damage. (**A**) Secreted human PGE_2_ in an MSC culture medium was determined using the ELISA. MSCs on each surface were treated with 100 ng/mL of IFN-γ for 24 h. 100 μM of indomethacin (marked as Indo) was treated with IFN-γ at the same time (*n* = 4 independent experiment; one-way ANOVA, * *p* < 0.05, and **** *p* < 0.0001). Error bars represent ±S.E.M. (**B**) At 6 h after sepsis was induced, MSCs for each condition were injected through the tail vein. Mice were assessed every day for up to 5 days. Percent survival was determined for each group and presented in the Kaplan–Meier format. (*n* = 10/group).

Although the primed MSCs on both surfaces protected against liver damage from polymicrobial sepsis, and provided that the SF nanofibers more effectively inhibited the acute plasma IL-6 and TNF-α levels, we hypothesized that the expression levels of secreted factors from primed MSCs between surfaces might differ. A few studies have reported that MSCs secrete soluble factors, such as transforming growth factor-β (TGF-β), prostaglandin E_2_ (PGE_2_), nitric oxide (NO), and indoleamine 2,3-dioxygenase (IDO), which can suppress activated T cells and regulate inflammatory responses [21,22,23]. A recent study also revealed that PGE_2_ secreted by MSCs protects against liver failure [24]. Thus, the level of PGE_2_ secreted by primed MSCs on each surface was analyzed using the ELISA. Thus, human MSCs were plated either on the control surface or on the SF nanofibers via incubation with IFN-γ. Although the SF nanofibers exhibited higher levels of PGE_2_ secretion from primed MSCs, Figure 5A shows human IFN-γ-treatment-induced PGE_2_ secretion from MSCs on both surfaces. Interestingly, the control surface and the SF nanofibers did not show a difference in PGE_2_ secretion when IFN-γ was not treated. In addition, indomethacin, a selective inhibitor of COX-2, which is a key enzyme for PGE_2_ production, completely blocked the secretion of PGE_2_ from primed MSCs on both surfaces, confirming that PGE_2_ secretion entirely depends on COX-2 expression, which is consistent with our previous study results, showing that the SF nanofibers enhanced COX-2 expression in MSCs [13]. Finally, primed MSCs with abrogated PGE_2_ secretion on the SF nanofibers failed to attenuate the mortality and morbidity of septic mice (Figure 5B). Overall, the SF nanofibers enhance the capability of MSCs to produce PGE_2_ and provide a culture platform for effective cell therapy.

## 4. Discussion

Sepsis is defined as a “systemic illness caused by the microbial invasion of normally sterile parts of the body”, and still has no cure despite improvements in the management of infection and organ damage [25]. MSCs have been postulated as a potential treatment for sepsis, owing to their immunomodulatory and anti-inflammatory properties. Many preclinical and clinical trials using MSCs have reported that they are safe and effective for treating sepsis [26,27]. However, several problems still exist in the translation of these cells to the clinical use. One of these problems is the number of cells required for MSC therapy.

Our study demonstrated that the SF nanofibers improved the anti-inflammatory function of MSCs preconditioned with IFN-γ. MSCs on the SF nanofibers significantly reduced the plasma IL-6 and TNF-α levels compared to MSCs on the control surface or vehicle-only groups within 24 h after injection (Figure 3), demonstrating the effective suppression of systemic inflammation in septic mice. The increase in plasma levels of inflammatory cytokines, such as IL-6 or TNF-α, is proportional to the severity of sepsis, and correlates with mortality [28]. Moreover, the strong prognostic value of IL-6 has been used to determine lethality in a polymicrobial sepsis model [29,30]. In this study, MSCs on the SF nanofibers significantly abrogated the massive increase in plasma IL-6 within 24 h, supporting the increase in survival rate compared to the vehicle group or the MSCs on the control surface (Figure 5B). Park et al. [31] demonstrated that nanovesicles (NVs) secreted from MSCs successfully abrogated the inflammatory response in mice with bacteria-induced sepsis. The proinflammatory cytokines IL-6 and TNF-α were significantly suppressed by NVs, and the anti-inflammatory cytokine IL-10 was highly enhanced by NV injection. NVs contain various NV proteins related to host defense/immunity responses, including syndecan-4, CD109, alpha-2-macroglobulin, and programmed cell death 1 ligand. These paracrine effects of MSCs are considered promising tools for treating inflammatory diseases. In some reports, plasma IL-6 levels after sepsis are essential mediators of mortality and morbidity due to sepsis in mice and humans [32]. Thus, blocking IL-6 using anti-IL-6 antibodies successfully improved the CLP murine model’s survival rate [33], indicating that the SF nanofiber scaffolds potentiate the immunomodulatory function of MSCs, resulting in the reduction of plasma IL-6 levels during polymicrobial sepsis, and lowering mortality in septic mice.

In our previous study, SF nanofibers further increased the expression of IDO-1 in MSCs via IFN-γ priming [13], which might partially contribute to the enhanced anti-inflammatory and immunomodulatory properties of MSCs investigated in the current study. Tipnis et al. reported that the immunosuppressive activity of MSCs on the proliferation of T and NK cells could be mediated by the enhancement of IDO production in MSCs [34]. COX-2, a key enzyme that produces PGE_2_, was also found to be elevated in our previous study. Here, we showed that PGE_2_ secretion was highly enhanced in MSCs on the SF nanofibers rather than MSCs on the control surface (Figure 5A). In this regard, the high expression of COX-2 in our previous study seems to be correlated with elevated PGE_2_ secretion. Although the role of PGE_2_ secreted from MSCs needs to be estimated in a subsequent study, we found that the COX-2–PGE_2_ axis plays a key role in protecting mice from sepsis, because indomethacin-treated MSCs completely failed to rescue septic mice (Figure 5B). Moreover, reduced PGE_2_ production seems to be critical for regulating MSC hypoimmunogenicity [35]. Overall, the SF nanofibers provide a beneficial microenvironment for MSCs to enhance their immunomodulatory and anti-inflammatory properties as a culture platform for cell therapy. In contrast to the acute response (24 h after sepsis) in this study, the chronic response at 5 days after MSC injection did not differ between the control and SF nanofiber surfaces. MSCs on both surfaces effectively reduced HO-1 and COX-2 expression and protected against tissue damage in the liver 5 days after sepsis induction. Some reports have indicated that intravenously infused MSCs are short-lived, and are primarily trapped in the lungs 1 h after infusion [36]. In addition, 24 h after MSC infusion, most MSCs died and were detected in the liver. Thus, altered tissue damage or protein expression in the liver might rarely be detectable between MSCs on the control surface and those on the SF nanofiber surface at 5 days after sepsis induction.

Silk is a natural composite fiber and a well-known biomaterial that possesses noteworthy biocompatibility and low antigenicity. In our results, SF nanofibers contained relatively high levels of amine groups (12.74%) on their surfaces (Figure 1C). A material surface containing an amine group (–NH_2_) allows cells, including MSCs, to enhance their attachment [37,38], which affects MSC function and activity [39]. Cellular adhesion associated with chemical groups, such as amine (–NH_2_) or carboxyl (–COOH), is attributed to electrostatic interactions between the cells and chemical groups on the material surface, rather than by specific receptor-mediated cell adhesion, including extracellular matrix or RGD peptide [40]. Nevertheless, an appropriate adhesion strength is necessary to initiate cell proliferation and cellular function. However, a very high adhesion strength, induced by a wide-spreading area or by the large focal adhesion plaques associated with the well-developed actin cytoskeleton, can interrupt cell proliferation, owing to the disassembly of the cellular structure before mitosis [41]. However, our previous study indicated that the SF nanofibers did not alter the cellular proliferation rate compared to the glass surface, whereas the migration speed of MSCs on the SF nanofibers was significantly higher than that on the control surface [13], suggesting that the cellular adhesion force with the SF nanofibers seems to be of appropriate strength, and that the SF nanofiber scaffolds provide a beneficial microenvironment for MSC culture. Taken together, the SF nanofiber scaffolds increase the anti-inflammatory ability of MSCs compared to regular surfaces, which potentiates the efficiency of MSC therapy. In addition, the SF nanofiber scaffolds can be utilized as a cost-effective culture platform to improve MSC therapy efficacy.

## 5. Conclusions

In this study, the SF nanofibers increased the immunomodulatory and anti-inflammatory functions of primed MSCs compared to the control surface when supplied as a culture scaffold. The MSCs on the SF nanofibers significantly reduced plasma IL-6 and TNF-α levels, which were increased by polymicrobial sepsis induction within 24 h. Although the SF nanofibers did not affect chronic inflammation or tissue damage in the liver, both MSC therapies reduced HO-1 and COX-2 expression in the livers of septic mice compared to the vehicle-treated group. Because acute IL-6 levels correspond to mortality and morbidity in sepsis, inhibiting plasma IL-6 using MSCs on the SF nanofibers contributed to the enhanced survival rate of septic mice. In addition, the SF nanofibers elevated PGE_2_ secretion through COX-2 expression from primed MSCs, compared to the control surface. Overall, the SF nanofibers provide a beneficial microenvironment for MSC cultivation, and enhance the immunomodulatory function of MSCs, which improves the efficiency of MSC therapy.

## Supplementary Materials

The following are available online at https://www.mdpi.com/article/10.3390/polym13091433/s1, Figure S1: The FTIR graph of SF nanofibers.

## References

[1] Singer M., Deutschman C.S., Seymour C.W., Shankar-Hari M., Annane D., Bauer M., Bellomo R., Bernard G.R., Chiche J.D., Coopersmith C.M. (2016). The Third International Consensus Definitions for Sepsis and Septic Shock (Sepsis-3). JAMA.

[2] Ayala A., Venet M.P.F., Lomas-Neira J., Swan R., Chung C.S. (2008). Apoptosis in Sepsis: Mechanisms, Clinical Impact and Potential Therapeutic Targets. Curr. Pharm. Des..

[3] Dombrovskiy V.Y., Martin A.A., Sunderram J., Paz H.L. (2007). Rapid Increase in Hospitalization and Mortality Rates for Severe Sepsis in the United States: A Trend Analysis from 1993 to 2003. Crit. Care Med..

[4] Melamed A., Sorvillo F.J. (2009). The Burden of Sepsis-Associated Mortality in the United States from 1999 to 2005: An Analysis of Multiple-Cause-of-Death Data. Crit. Care.

[5] Ho M.S., Mei S.H., Stewart D.J. (2015). The Immunomodulatory and Therapeutic Effects of Mesenchymal Stromal Cells for Acute Lung Injury and Sepsis. J. Cell Physiol..

[6] Lee H.J., Kim W.Y. (2020). Mesenchymal Stromal Cell Application as an Emerging Translational Medicine for Acute Respiratory Distress Syndrome. Ann. Tranl. Med..

[7] Wu J., Wang Y., Li L. (2017). Functional Significance of Exosomes Applied in Sepsis: A Novel Approach to Therapy. Biochim. Biophys. Acta.

[8] Li W., Chen W., Huang S., Yao G., Tang X., Sun L. (2020). Mesenchymal Stem Cells Prevent Overwhelming Inflammation and Reduce Infection Severity Via Recruiting Cxcr3+ Regulatory T Cells. Clin. Transl. Immunol..

[9] Nemeth K., Mayer B., Mezey E. (2010). Modulation of Bone Marrow Stromal Cell Functions in Infectious Disease by Toll-Like Receptor Ligands. J. Mol. Med..

[10] Dominici M., Le Blanc K., Mueller I., Slaper-Cortenbach I., Marini F., Krause D., Deans R., Keating A., Prockop D., Horwitz E. (2006). Minimal Criteria for Defining Multipotent Mesenchymal Stromal Cells. The International Society for Cellular Therapy Position Statement. Cytotherapy.

[11] Kobabu S., Lowery J.W., Jimi E. (2016). Cell Fate and Differentiation of Bone Marrow Mesenchymal Stem Cells. Stem. Cell Int..

[12] Diaz M.F., Vaidya A.B., Evans S.M., Lee H.J., Aertker B.M., Alexander A.J., Price K.M., Ozuna J.A., Liao G.P., Aroom K.R. (2017). Biomechanical Forces Promote Immune Regulatory Function of Bone Marrow Mesenchymal Stromal Cells. Stem. Cells.

[13] Kim O.H., Yoon O.J., Lee H.J. (2019). Silk Fibroin Scaffolds Potentiate Immunomodulatory Function of Human Mesenchymal Stromal Cells. Biochem. Biophys. Res. Commun..

[14] Kapoor S., Kundu S.C. (2016). Silk Protein-Based Hydrogels: Promising Advanced Materials for Biomedical Applications. Acta Biomater..

[15] Sapru S., Das S., Mandal M., Ghosh A.K., Kundu S.C. (2018). Prospects of Nonmulberry Silk Protein Sericin-Based Nanofibrous Matrices for Wound Healing—In Vitro and in Vivo Investigations. Acta Biomater..

[16] Aronoff D.M. (2012). Cyclooxygenase Inhibition in Sepsis: Is There Life after Death?. Mediat. Inflamm..

[17] Khatri M., Hussain N., Ghazali S.E., Yamamoto T., Kobayashi S., Khatri Z., Ahmed F., Kim I.S. (2020). Ultrasonic Assisted Dyeing of Silk Fbroin Nanofbers: An Energy Efcient Coloration at Room Temperature. Appl. Nanosci..

[18] Shao J., Liu J., Zheng J., Carr C.M. (2002). X-Ray Photoelectron Spectroscopic Study of Silk Fibroin Surface. Polym. Int..

[19] Nasim A., Mahdi N., Mohammad H.K. (2010). Structural Characterization and Mechanical Properties of Electrospun Silk Fibroin Nanofiber Mats. Polym. Sci..

[20] Leila S., Mahdi N. (2016). Electrospun Silk Fibroin Nanofibers with Improved Surface Texture. J. Text. Polym..

[21] Meisel R., Zibert A., Laryea M., Göbel U., Däubener W., Dilloo D. (2004). Human Bone Marrow Stromal Cells Inhibit Allogeneic T-Cell Responses by Indoleamine 2,3-Dioxygenase-Mediated Tryptophan Degradation. Blood.

[22] Spaggiari G.M., Capobianco A., Abdelrazik H., Becchetti F., Mingari M.C., Moretta L. (2008). Mesenchymal Stem Cells Inhibit Natural Killer-Cell Proliferation, Cytotoxicity, and Cytokine Production: Role of Indoleamine 2,3-Dioxygenase and Prostaglandin E2. Blood.

[23] Wang C., Chen J., Sun L., Liu Y. (2014). Tgf-Beta Signaling-Dependent Alleviation of Dextran Sulfate Sodium-Induced Colitis by Mesenchymal Stem Cell Transplantation. Mol. Biol. Rep..

[24] Liu Y., Ren H., Wang J., Yang F., Li J., Zhou Y., Yuan X., Zhu W., Shi X. (2018). Prostaglandin E2 Secreted by Mesenchymal Stem Cells Protects against Acute Liver Failure Via Enhancing Hepatocyte Proliferation. FASEB J..

[25] Lever A., Mackenzie I. (2007). Sepsis: Definition, Epidemiology, and Diagnosis. BMJ.

[26] McIntyre L.A., Stewart D.J., Mei S.H.J., Courtman D., Watpool I., Granton J., Marshall J., dos Santos C., Walley K.R., Winston B.W. (2018). Cellular Immunotherapy for Septic Shock. A Phase I Clinical Trial. Am. J. Respir. Crit. Care Med..

[27] Sun X.Y., Ding X.F., Liang H.Y., Zhang X.Y., Liu S.H., Han B., Duan X.G., Sun T.W. (2020). Efficacy of Mesenchymal Stem Cell Therapy for Sepsis: A Meta-Analysis of Preclinical Studies. Stem. Cell Res. Ther..

[28] Oberholzer A., Oberholzer C., Moldawer L.L. (2001). Sepsis Syndromes: Understanding the Role of Innate and Acquired Immunity. Shock.

[29] Ebong S.J., Call D.R., Bolgos G., Newcomb D.E., Granger J.I., O’Reilly M., Remick D.G. (1999). Immunopathologic Alterations in Murine Models of Sepsis of Increasing Severity. Infect. Immun..

[30] Remick D.G., Bolgos G.R., Siddiqui J., Shin J., Nemzek J.A. (2002). Six at Six: Interleulin-6 Measured 6 H after the Initiation of Sepsis Predicts Mortality over 3 Days. Shock.

[31] Park K.S., Svennerholm K., Shelke G.V., Bandeira E., Lässer C., Jang S.C., Chandode R., Gribonika I., Lötvall J. (2019). Mesenchymal Stromal Cell-Derived Nanovesicles Ameliorate Bacterial Outer Membrane Vesicle-Induced Sepsis Via Il-10. Stem Cell Res. Therapy.

[32] Hack C.E., De Groot E.R., Felt-Bersma R.J., Nuijens J.H., Strack Van Schijndel R.J., Eerenberg-Belmer A.J., Thijs L.G., Aarden L.A. (1989). Increased Plasma Levels of Interleukin-6 in Sepsis. Blood.

[33] Riedemann N.C., Neff T.A., Guo R.F., Bernacki K.D., Laudes I.J., Sarma J.V., Lambris J.D., Ward P.A. (2003). Rotective Effects of Il-6 Blockade in Sepsis Are Linked to Reduced C5a Receptor Expression. J. Immunol..

[34] Tipnis S., Viswanathan C., Majumdar A.S. (2010). Immunosuppressive Properties of Human Umbilical Cord-Derived Mesenchymal Stem Cells: Role of B7-H1 and Ido. Immunol. Cell Biol..

[35] Saparov A., Ogay V., Nurgozhin T., Jumabay M., Chen W.C.W. (2016). Preconditioning of Human Mesenchymal Stem Cells to Enhance Their Regulation of the Immune Response. Stem. Cell Int..

[36] Eggenhofer E., Benseler V., Kroemer A., Popp F.C., Geissler E.K., Schlitt H.J., Baan C.C., Dahlke M.H., Hoogduijn M.J. (2012). Mesenchymal Stem Cells Are Short-Lived and Do Not Migrate Beyond the Lungs after Intravenous Infusions. Front. Immunol..

[37] Cao B., Peng Y., Liu X., Ding J. (2017). Effects of Functional Groups of Materials on Nonspecific Adhesion and Chondrogenic Induction of Mesenchymal Stem Cells on Free and Micropatterned Surfaces. ACS Appl. Mater. Interfaces.

[38] Noronha N.C., Mizukami A., Caliári-Oliveira C., Cominal J.G., Rocha J.L.M., Covas D.T., Swiech K., Malmegrim K.C.R. (2019). Priming Approaches to Improve the Efficacy of Mesenchymal Stromal Cell-Based Therapies. Stem Cell Res. Ther..

[39] Caliari S.R., Vega S.L., Kwon M., Soulas E.M., Burdick J.A. (2016). Dimensionality and Spreading Influence Msc Yap/Taz Signaling in Hydrogel Environments. Biomaterials.

[40] Černochová P., Blahová L., Medalová J., Nečas N., Michlíček M., Kaushik P., Přibyl J., Bartošíková J., Manakhov A., Bačáková L. (2020). Cell Type Specific Adhesion to Surfaces Functionalised by Amine Plasma Polymers. Sci. Rep..

[41] Bacakova L., Filova E., Parizek M., Ruml T., Svorcik V. (2011). Modulation of Cell Adhesion, Proliferation and Differentiation on Materials Designed for Body Implants. Biotechnol. Adv..

