# Supplementary material for: Bone Marrow Mesenchymal Stromal Cells on Silk Fibroin Scaffolds to Attenuate Polymicrobial Sepsis Induced by Cecal Ligation and Puncture
Source: Polymers (Basel). 2021 Apr 29;13(9):1433. doi: 10.3390/polym13091433 (PMC8125697; doi:10.3390/polym13091433)
Supplement: Supplementary file 1 [file polymers-13-01433-s001.zip › polymers-1183305-supplementary.pdf]

polymers

MDPI

Supplementary Materials

Bone Marrow Mesenchymal Stromal Cells on Silk Fibroin Scaffolds to Attenuate Polymicrobial Sepsis Induced by Cecal Ligation and Puncture

Ok-Hyeon Kim [1,2], Jun Hyung Park [1,2], Jong In Son [1], Ok-Ja Yoon [3,*] and Hyun Jung Lee [1,2,*]

[1] Department of Anatomy and Cell Biology, College of Medicine, Chung-Ang University, Seoul 06974, Korea; ssimba315@cau.ac.kr (O.-H.K.); june8902@cau.ac.kr (J.H.P.); jison@cau.ac.kr (J.I.S.)

[2] Department of Global Innovative Drugs, Graduate School of Chung-Ang University, Seoul 06974, Korea

[3] Da Vinci College of General Education, Chung-Ang University, Seoul 06974, Korea

* Correspondence: yokk777@cau.ac.kr (O.-J.Y.); pluto38@cau.ac.kr (H.J.L.); Tel.: +82-2-820-6769 (O.-J.Y.); Tel.: +82-2-820-5434 (H.J.L.)

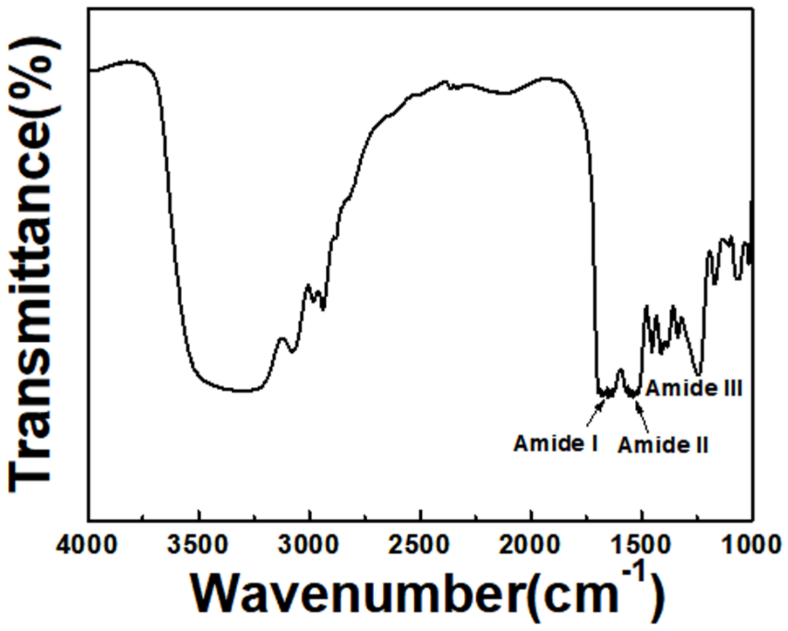

Figure S1. The FTIR graph of SF nanofibers.

Chemical and structural analyses of the SF nanofibers were performed using Fourier-transform infrared spectrophotometry. FTIR analysis was performed with the SF film having identical concentration as the SF nanofibers.
